# Why Medical Schools Should Embrace Wikipedia: Final-Year Medical Student Contributions to Wikipedia Articles for Academic Credit at One School

**DOI:** 10.1097/ACM.0000000000001381

**Published:** 2016-09-13

**Authors:** Amin Azzam, David Bresler, Armando Leon, Lauren Maggio, Evans Whitaker, James Heilman, Jake Orlowitz, Valerie Swisher, Lane Rasberry, Kingsley Otoide, Fred Trotter, Will Ross, Jack D. McCue

**Affiliations:** **A. Azzam** is associate clinical professor, Department of Psychiatry, University of California, San Francisco, San Francisco, California.; **D. Bresler** is resident physician, Department of Psychiatry, University of California, Los Angeles, Los Angeles, California.; **A. Leon** is resident physician, San Mateo County Psychiatry Residency Training Program, San Mateo, California.; **L. Maggio** is associate professor, Department of Medicine, Uniformed Services University of the Health Sciences, Bethesda, Maryland.; **E. Whitaker** is education and information consultant for medicine, Library and Center for Knowledge Management, University of California, San Francisco, San Francisco, California.; **J. Heilman** is clinical assistant professor, Department of Emergency Medicine, University of British Columbia, Vancouver, British Columbia, Canada.; **J. Orlowitz** is head, Wikipedia Library, Wikimedia Foundation, San Francisco, California.; **V. Swisher** is board member, Translators Without Borders, and chief executive officer, Content Rules, Inc., Los Gatos, California.; **L. Rasberry** is Wikipedian in residence, Consumer Reports, Yonkers, New York.; **K. Otoide** is cofounder, Insp-i.com, Lagos, Nigeria.; **F. Trotter** is data journalist, DocGraph, Houston, Texas.; **W. Ross** is project manager, Mendocino Informatics, Inc., Mendocino, California.; **J.D. McCue** is emeritus professor of medicine, Department of Medicine, University of California, San Francisco, San Francisco, California.

## Abstract

**Problem:**

Most medical students use Wikipedia as an information source, yet medical schools do not train students to improve Wikipedia or use it critically.

**Approach:**

Between November 2013 and November 2015, the authors offered fourth-year medical students a credit-bearing course to edit Wikipedia. The course was designed, delivered, and evaluated by faculty, medical librarians, and personnel from WikiProject Medicine, Wikipedia Education Foundation, and Translators Without Borders. The authors assessed the effect of the students’ edits on Wikipedia’s content, the effect of the course on student participants, and readership of students’ chosen articles.

**Outcomes:**

Forty-three enrolled students made 1,528 edits (average 36/student), contributing 493,994 content bytes (average 11,488/student). They added higher-quality and removed lower-quality sources for a net addition of 274 references (average 6/student). As of July 2016, none of the contributions of the first 28 students (2013, 2014) have been reversed or vandalized. Students discovered a tension between comprehensiveness and readability/translatability, yet readability of most articles increased. Students felt they improved their articles, enjoyed giving back “specifically to Wikipedia,” and broadened their sense of physician responsibilities in the socially networked information era. During only the “active editing months,” Wikipedia traffic statistics indicate that the 43 articles were collectively viewed 1,116,065 times. Subsequent to students’ efforts, these articles have been viewed nearly 22 million times.

**Next Steps:**

If other schools replicate and improve on this initiative, future multi-institution studies could more accurately measure the effect of medical students on Wikipedia, and vice versa.

## Abstract 2

Supplemental Digital Content is available in the text.

## Problem

The rise and spread of Internet accessibility has created an unprecedented resource for the dissemination of medical information, as well as an invaluable tool for health care providers and the general public alike. At the same time, opportunities for the rapid spread of misinformation or the misinterpretation of medical facts have never been greater. Clinicians must sometimes gently redirect patients who are convinced that they have some rare disease they have read about on the Internet. Although medical educators typically train students to address patient misperceptions in clinical practice, they are not leveraging clinician and medical student knowledge to improve the quality of the information patients and others find online.

Wikipedia is a free, online, multilingual encyclopedia that is continually and collaboratively created. Anyone with an Internet connection can edit its articles. One of the most frequently visited Web sites worldwide, it is among the leading sources of health information for medical professionals and health care consumers alike.^1^ At the end of 2013, Wikipedia’s medical content included over 155,000 articles written in 255 languages, supported by more than 950,000 references.^2^ Despite its increasingly prevalent use as a medical information resource in clinical practice, clinical instructors and faculty members often dissuade medical students from using Wikipedia, citing concern for its perceived inaccuracies and lack of traditional editorial controls.^3^

Although physicians and medical students are encouraged to contribute to traditional sources of medical knowledge (e.g., textbooks and journals), the idea of benefiting from active contribution to crowd-sourced resources such as Wikipedia remains the perspective of a minority or fringe group within the academic medicine community.

We believe that *not* contributing to crowd-sourced resources represents a lost opportunity for enriching medical students’ learning and for disseminating more accurate, up-to-date medical information to Wikipedia’s readers worldwide.

## Approach

We created what is, to our knowledge, the first formal medical school course worldwide through which medical students actively work to improve Wikipedia’s health-related articles. We expected enrolled students to hone their information retrieval and assessment skills,^4^ practice communicating medical knowledge to an exceptionally broad global audience,^2^ and expand their sense of health care providers’ roles in the Internet age. We designed our course with assistance from *WikiProject Medicine*, a volunteer group of experienced Wikipedia editors who seek to ensure that the general public and health care professionals have access to free, current, accurate, and understandable medical information in their own language.

Thus far, we have run the course four times: November 2013, April 2014, November 2014, and November 2015. Each cycle has begun with a two-day orientation (Table 1) during which we introduce students to Wikipedia’s editorial tools, style, and standards. Additional didactic activities include reviewing guidelines for writing simplified English and strategies for locating and evaluating source material quality.

**Table 1:**
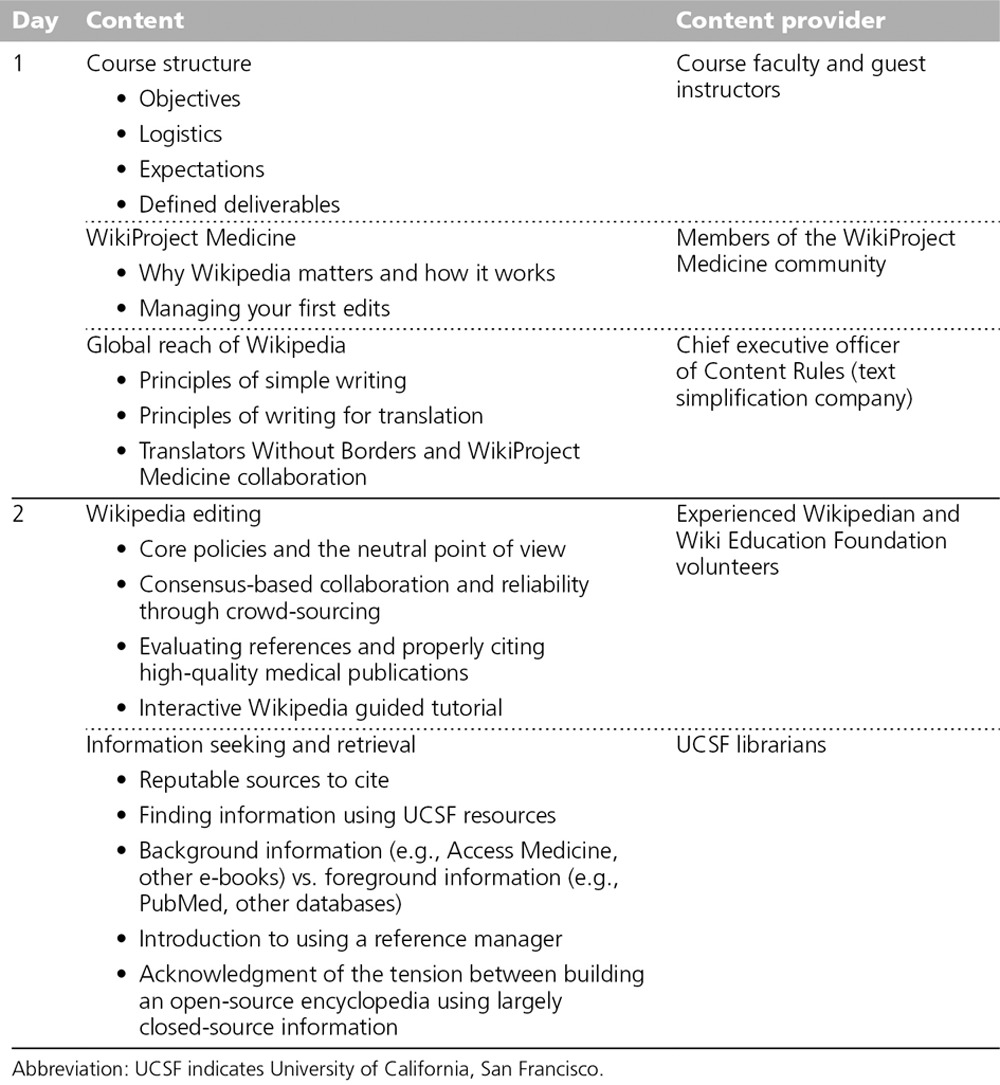
Instructional Content During Initial Two-Day Orientation Session of UCSF Wikipedia-Editing Course

All students establish their own Wikipedia user accounts and choose a single health-related article to edit over the remainder of the course. *WikiProject Medicine* maintains a list of the most frequently accessed health-related Wikipedia articles, ranked by importance, and graded according to an article quality scheme that is applied to most Wikipedia Projects.^5^ We encourage (but do not require) students to select a “top-” or “high-importance” article that has also been tagged as needing quality improvement. At the beginning of the course, students receive custom reports for their chosen articles that contain both a list of grammar and style errors and a quantitative readability score. These Acrolinx (San Jose, California) reports use specialized natural language processing software, which considers sentence length and over 100 grammar and style rules.

Detailed course structure and materials are publicly available under free license on our course Wikipedia page.^6^ As we are not always certain, a priori, how much students can accomplish in a month, we encourage them to improve their selected articles “as much as feasible.” Thus far, students have completed the majority of their editing work independently. Instructors (A.A. and J.H.) and medical librarians (E.W. and L.M.) provide intermittent encouragement via e-mail, hold weekly office hours, and schedule consultation as needed. At the end of the course, faculty provide students access to custom-built tools that allow comparison of summary statistics of each article on the first versus last day of the course. While presenting the final versions of their Wikipedia pages, all students share highlights of their accomplishments and challenges, describe lessons learned, and provide feedback for future iterations of the course.

This report summarizes the outcomes of our pilot initiative at the University of California, San Francisco (UCSF). The UCSF institutional review board reviewed this research and deemed it exempt from formal review.

### Effect on Wikipedia articles

We compared the state of the Wikipedia articles before and after student editing using several approaches. Two UCSF-affiliated physicians (E.W. and J.D.M.) provided independent subjective ratings of article changes and reconciled their impressions by consensus. Wikipedians with experience rating medical articles but unaffiliated with the elective received blinded unedited and edited versions of each article to evaluate using the general WikiProject article-grading rubric. We obtained changes in word, paragraph, and citation counts using Wikipedia’s analysis tools. Finally, we quantitatively assessed each article’s quality, readability, and translatability by generating postcourse Acrolinx reports.

Conveniently, all Wikipedia edits are saved in perpetuity. This archiving permits easy comparison across article versions over time. Because students edited their articles via their Wikipedia user accounts, we are able to analyze the changes (to words, paragraphs, and citations) made directly by our students. However, because anyone at any time can edit Wikipedia articles, all other metrics comparing the state of an article at the beginning and end of the course inevitably include changes made by other (nonstudent) editors.

### Effect on medical students

At the midpoint of each course, three authors (A.A., E.W., L.M.) conducted one-on-one semistructured interviews with all enrolled students, asking nine questions about students’ evolving impressions of the work of editing Wikipedia. Additionally, for each cycle we conducted a single end-of-course focus group with all actively enrolled students. All interviews and focus group sessions were audio recorded and transcribed. Several authors (A.A., A.L., L.M., K.O.) coded the emerging themes independently and then generated an overall theme list by consensus. Although we did not formally member check the final themes, we informed students that their comments would be deidentified and analyzed in aggregate for scholarly dissemination.

## Outcomes

### Effect on Wikipedia articles

During their participation in the course, the 43 students who enrolled (2013–2015) each edited a different Wikipedia article. Collectively, the students made 1,528 edits (average 36/student), contributing 493,994 content bytes (average 11,488/student). They added higher-quality and removed lower-quality sources for a net addition of 274 references (average 6/student).

Additionally, we conducted a detailed analysis of the first three cycles of the course (November 2013–November 2014), during which 28 students enrolled and completed the course. Supplemental Digital Appendix 1 (http://links.lww.com/ACADMED/A386) provides a representative example of students’ work. Subjective quality improvement ratings (“very improved,” “improved,” “unimproved,” “worse,” “much worse”) determined by consensus between two UCSF physicians revealed that overall quality of 14 articles was “very improved,” 12 articles “improved,” and 2 articles “unimproved.” Using the WikiProject article-grading scheme (stub, start, C, B, good, featured), all articles either improved in rating or remained unchanged; no articles declined in rating. Eight articles were initially tagged “Stub” or “Start” quality. Of these, 3 moved up two levels, 4 increased one level, and 1 remained unchanged. Fifteen articles were initially “C” quality. Of these, 4 increased one level, and 11 remained unchanged. Five articles were initially “B” quality, and all 5 of these remained unchanged. Table 2 summarizes the effect on Wikipedia article quality.

**Table 2:**
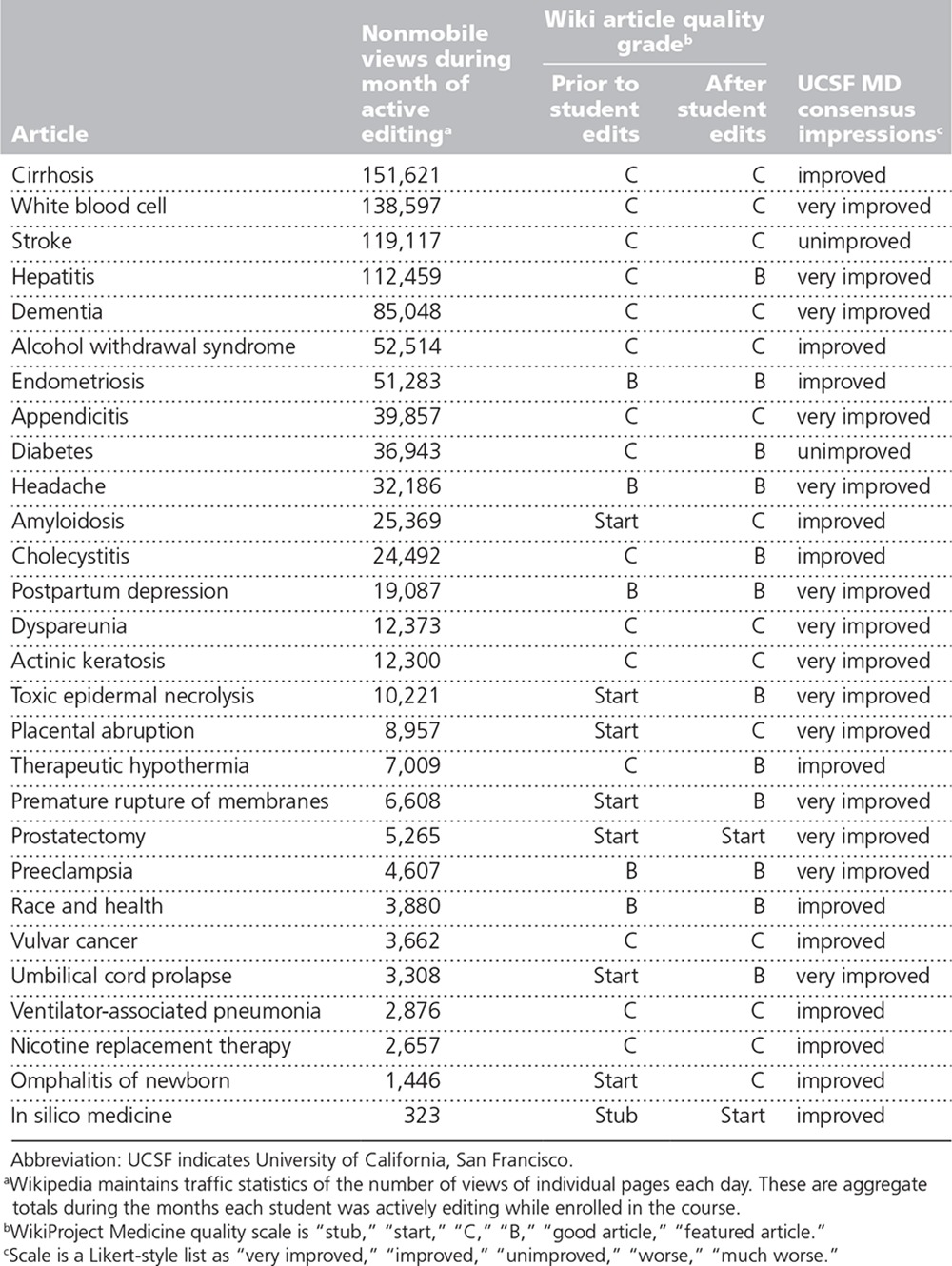
Summary of Effect of UCSF Medical Students’ Editing on Wikipedia Article Quality

The quantitative review of individual student contributions revealed that 19 of these 28 articles had substantial changes to at least half of the sections. The average net change in number of paragraphs during the course was +7 (range −13 to +30). Three articles had a net deletion of words (−413 average; range −40 to −740). The remaining 25 articles added a total of 7,510 words (average net +300 per article; range +29 to +3,649). The number of citations increased in 24 articles. Among these, we noted an average net increase of 5.7 references per article (37.8 before to 43.5 after).

Natural language processing software showed modest but consistent average improvements across all scoring categories (all graded on a 100-point scale) for all 28 articles. In particular, overall quality-of-writing score improved in 23 articles (mean score increased from 61.1 to 62.9), style score improved in 22 articles (mean score increased from 32.0 to 34.7), readability score improved in 22 articles (mean score increased from 64.9 to 66.5), and grammar score improved in 20 articles (mean score increased from 90.8 to 91.6).

### Effect on medical students

The transcription and coding of interviews and focus groups with the first 28 students revealed several themes (see Table 3). Many students found researching and editing the articles to be more challenging than predicted. Some students cited the opportunity to give back to Wikipedia as a reason for enrolling, but quickly realized the work included much more than just writing. Students discovered a tension between comprehensiveness and readability/translatability, as the need to simultaneously address both general population and medical professional audiences proved difficult. Students had not anticipated and were at times challenged by the collaborative nature of the work, which variously required a willingness to rewrite, reorganize, or remove large portions of others’ work. Students largely felt that these challenges were balanced by their perception of the utility and potential global significance of their efforts. Lastly, students deeply appreciated the independent nature of the course—allowing for flexibility and travel during their residency application process.

**Table 3:**
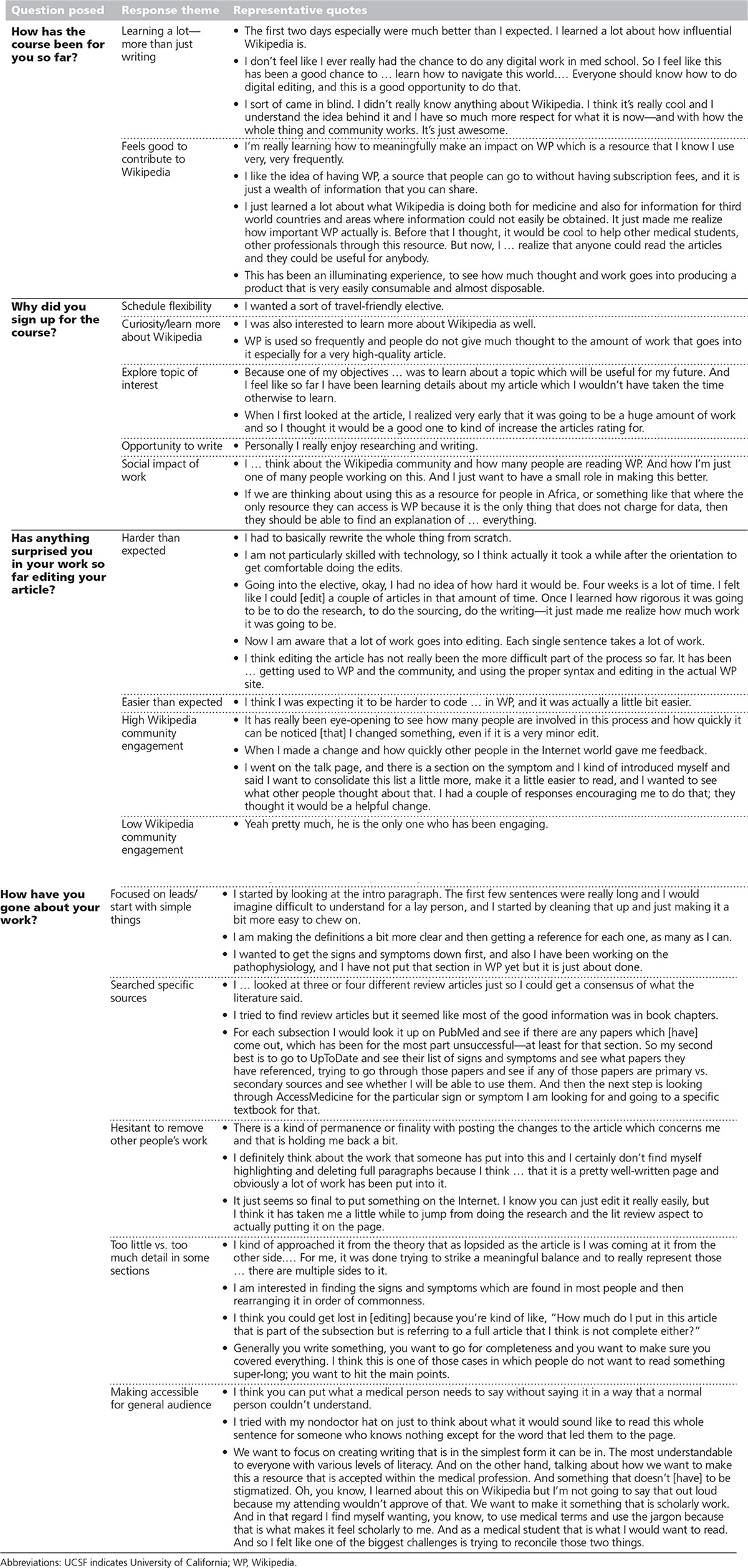
Summary of Effect of Wikipedia-Editing Course on UCSF Medical Students

## Discussion

### The changing role of the provider.

Students said that this elective widened their perceptions of being a health care provider in the modern age, especially in regard to Wikipedia. One of the more difficult facets of medical practice is effectively communicating complex health-related concepts. Although students have traditionally learned this skill “on the job,” our course directly fosters clear communication in a nonclinical setting. Students gained concrete experience explaining nuanced health information in a way that is accessible to the general public. In addition, students reported that they felt joining the active community of Wikipedia editors and providing accurate information to communities that do not have access to other resources were rewarding. Furthermore, as the medical community moves toward more interprofessional care of patients and increases its reliance on electronic health records, this asynchronous cross-disciplinary virtual collaboration mirrors the evolving experience of clinical care. Additionally, by commissioning experienced Wikipedia editors as well as UCSF-affiliated physician–volunteers as participants in the course, we leveraged resources that have been untapped and/or underused in medical schools.

### Meaningful and lasting improvements.

The students’ positive feedback suggests that we have tapped a historically underdeveloped and potentially transformative opportunity to improve the curriculum. We believe that the wide variability across students’ accomplishments in text and citation changes indicated not only each student’s individual approach to editing but also, likely, the varying initial quality of the articles. In course feedback sessions, students frequently cited the flexibility to develop their own approach to editing the articles as a major strength of the course; however, this flexibility, paired with the variability in initial article quality, introduced challenges in evaluating students’ efforts. For example, an article might be substantively improved through the removal of content, ultimately resulting in a decreased word count. Regardless, through July 2016, *all* of our students’ contributions have persisted in Wikipedia—in several cases for over two years. Even marginal improvements thus can have a lasting impact.

According to Wikipedia’s traffic statistics, during only the month that the students were actively contributing, the 43 articles edited in 2013, 2014, and 2015 were collectively viewed 1,116,065 times. Since then, between the end date of each course and October 31, 2015 (an arbitrary end date), these 43 articles had been viewed in aggregate 12,865,783 times. This remarkable total does not even include views or access via mobile devices (we do not have specific reports from mobile devices). Extrapolating Wikipedia traffic ratios of mobile versus desktop computer access in October 2015,^2^ we estimate the overall views of these 43 articles since students finished editing until October 31, 2015, to be 21,992,791.

## Next Steps

### Evolving definition of scholarly work

As more journals move towards open access, and more health care professionals contribute to Wikipedia as the quintessential open resource, our students’ efforts raise interesting questions about the definition of what constitutes scholarly activity within medicine and, more broadly, all professions. How does the creation of a community resource differ from the use of a textbook written by experts in the field? Should contributions to a community resource be considered a form of scholarly activity? Could viewership serve as a metric, complementary to impact factor? If a Wikipedia article edited by a medical student garners over 100,000 views/month, might those edits constitute the greatest contribution to the medical literature in that student’s nascent career? Are the public reviews, offered by professionals (or professionals-in-training), on talk pages of Wikipedia articles, serving as a version of peer review? And as professionals join the community of Wikipedia editors and contributors, how do their articles and edits change the definitions of “peer” and especially “peer review”?

### Other metrics

In designing our first-of-its-kind course, we had no guidance on what outcome metrics to assess, so we made our best guesses, including counting visits to each student’s Wikipedia article. We are not suggesting that more people go to these Wikipedia pages *because* they were touched by medical students; rather, we are showing that these numbers are significantly higher than those who typically assess traditional course assignments. In hindsight, these metrics prove only what we all intuitively know—a lot of people go to Wikipedia for health information. These data put that conventional wisdom into staggering context. Still, other innovative metrics may help us assess students’ edited articles in the future.

### Future studies

Our preliminary data are from a relatively small number of students; future studies should examine the effects of greater numbers of medical students’ contributions to Wikipedia. Additional next steps might include systematically measuring if and how students use the collaborative tools incorporated into Wikipedia; how the course affects students’ ability to communicate difficult medical concepts, especially to patients; or how much students continue to contribute to Wikipedia after completing the course. In addition to helping better understand medical students’ impact on Wikipedia, these outcomes could provide better tools for evaluating student effort as well as for measuring the long-term influence of the course on the students.

Using Wikipedia’s internal metrics, our own independent qualitative grading criteria, and objective quantitative measures of readability, we have demonstrated that medical students are able to improve the quality of Wikipedia’s health-related articles during a monthlong course, simultaneously develop their skills as health care educators, and leave the course with a broadened understanding of their professional roles. Still, we continue refining our course, and had an additional 22 medical students enrolled during the 2015–2016 academic year. Though this remains a small number of students, we have developed a course model based on openly available resources that could be offered at other health professional training programs around the world. With the spread of similar courses,^7,8^ greater numbers of medical students contributing to Wikipedia could create opportunities for multi-institutional collaborative studies with larger sample sizes. These future collaborations would more accurately measure the potential effect of medical students’ contributions to Wikipedia—and vice versa.

The collaborative structure of Wikipedia creates an ideal platform for students across a range of health-related fields and geographic locations to work together to improve one of the most widely accessed repositories of health care information.^2^ The nonprofit organization Translators Without Borders is actively partnering with *WikiProject Medicine* to translate health-related articles to other language Wikipedias.^9^ As of July 2016, they have translated 637 English Wikipedia articles into 50 other languages. Furthermore, the Wikipedia Zero initiative now provides access to Wikipedia for free to over 600 million people in 57 developing countries via 75 mobile carriers.^10^ Given the broad-ranging readership of Wikipedia, the potential for the next generation of health providers—working as “digital contributors” and not merely “digital consumers”—to effect positive worldwide change in human health is immense.

## 

*Acknowledgments:* The authors wish to acknowledge Jaspreet Singh and Veronica Gonzalez for their valuable assistance in transcribing the interviews and focus groups; Claire Chen-Carter for Supplemental Digital Appendix 1; Eleanor Katari for copyediting advice; and of course, the 28 brave students who pioneered this elective.

## Supplementary Material

## References

[1] Aitken M Engaging patients through social media: Is healthcare ready for empowered and digitally demanding patients?. http://www.imshealth.com/files/web/IMSH%20Institute/Reports/Engaging%20Patients%20Through%20Social%20Media/IIHI_Social_Media_Report_2014.pdf.

[2] Heilman JM, West AG (2015). Wikipedia and medicine: Quantifying readership, editors, and the significance of natural language.. J Med Internet Res.

[3] Metcalfe D, Powell J (2011). Should doctors spurn Wikipedia?. J R Soc Med.

[4] Badgett RG, Moore M (2011). Are students able and willing to edit Wikipedia to learn components of evidence-based practice?. Kans J Med.

[5] Wikipedia WikiProject Medicine / Assessment.. https://en.wikipedia.org/wiki/Wikipedia:WikiProject_Medicine/Assessment.

[6] Wikipedia Wikipedia:WikiProject Medicine/UCSF.. http://en.wikipedia.org/wiki/Wikipedia:WikiProject_Medicine/UCSF.

[7] Wikipedia Wikipedia: WikiProject Medicine/ Icahn.. https://en.wikipedia.org/wiki/Wikipedia:WikiProject_Medicine/Icahn.

[8] Wikipedia Wikipedia WikiProject Medicine/ Tel Aviv University.. https://en.wikipedia.org/wiki/Wikipedia:WikiProject_Medicine/Tel_Aviv_University.

[9] Wikipedia Wikipedia:WikiProject Medicine/Translation Task Force.. http://en.wikipedia.org/wiki/Wikipedia:WikiProject_Medicine/Translation_task_force.

[10] Wikimedia Foundation Wikipedia Zero.. http://wikimediafoundation.org/wiki/Wikipedia_Zero.

