# Supplementary material for: Why Medical Schools Should Embrace Wikipedia: Final-Year Medical Student Contributions to Wikipedia Articles for Academic Credit at One School
Source: Acad Med. 2016 Sep 13;92(2):194–200. doi: 10.1097/ACM.0000000000001381 (PMC5265689; doi:10.1097/ACM.0000000000001381)
Supplement: Supplementary file 1 [file acm-92-194-s001.pdf]

Supplemental Digital Appendix 1

Example Wikipedia Article, Prior to and After UCSF Medical Student's Contributions

Prior

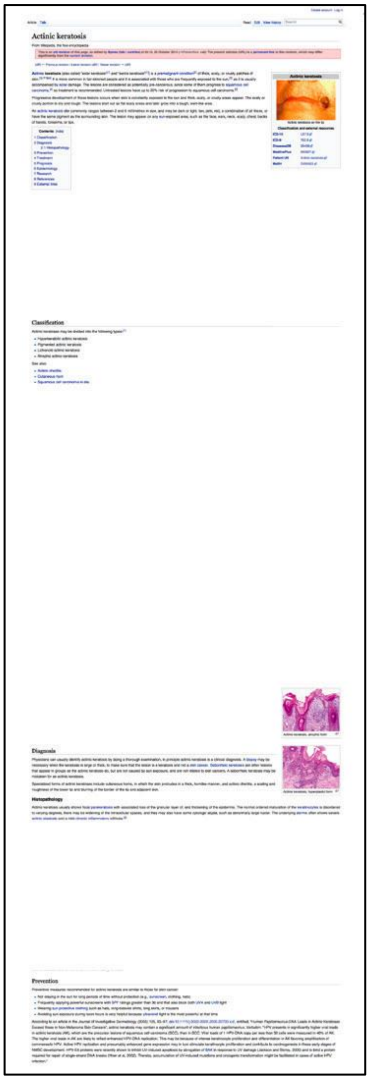

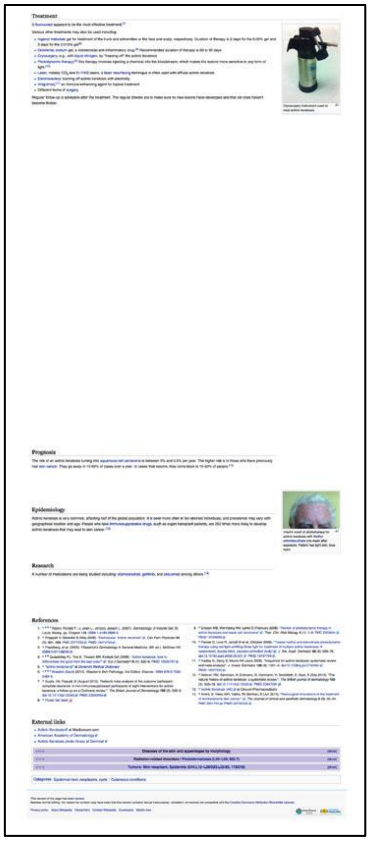

After

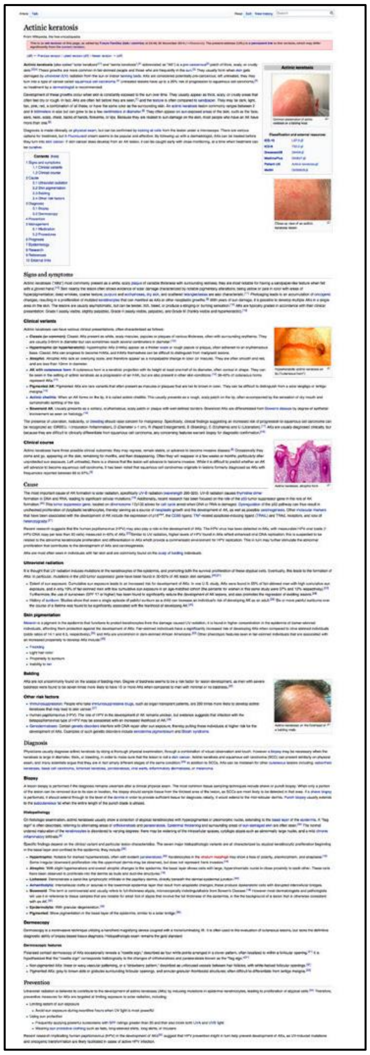

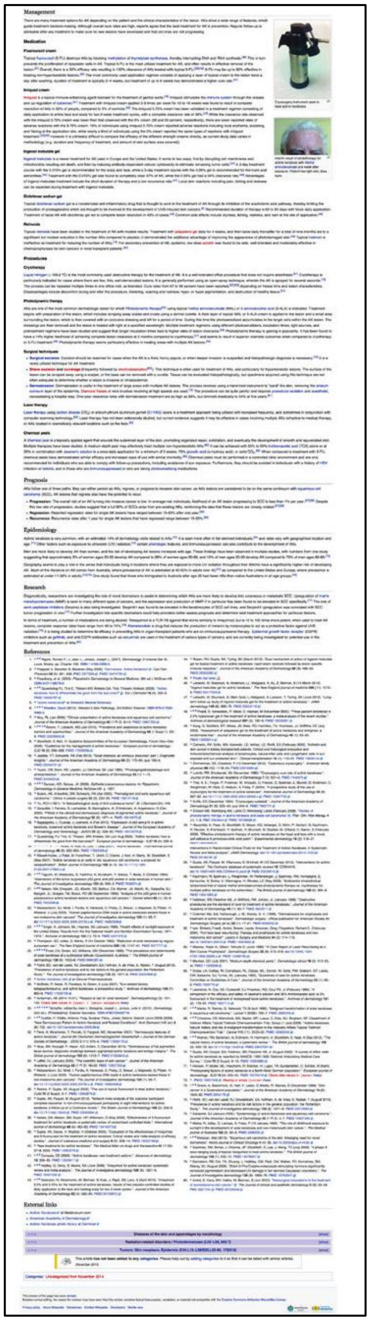

Copyright © by the Association of American Medical Colleges. Unauthorized reproduction is prohibited.

Close Up of Lead Portion of a Wikipedia Article Prior to UCSF Medical Student's First Contribution

Actinic keratosis

From Wikipedia, the free encyclopedia

This is an old revision of this page, as edited by Bjenks (talk | contribs) at 03:15, 25 October 2014 *(→Prevention: +wl)*. The present address (URL) is a permanent link to this revision, which may differ significantly from the current revision.

Actinic keratosis (also called "solar keratosis"[1] and "senile keratosis"[1]) is a premalignant condition[2] of thick, scaly, or crusty patches of skin.[3]:719[4] It is more common in fair-skinned people and it is associated with those who are frequently exposed to the sun,[5] as it is usually accompanied by solar damage. The lesions are considered as potentially pre-cancerous, since some of them progress to squamous cell carcinoma,[4] so treatment is recommended. Untreated lesions have up to 20% risk of progression to squamous cell carcinoma.[6]

Actinic keratosis

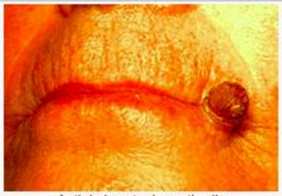

Actinic keratosis on the lip

Classification and external resources

ICD-10	L57.0
ICD-9	702.0
DiseasesDB	29438
MedlinePlus	000827
Patient UK	Actinic keratosis
MeSH	D055623

Progressive development of these lesions occurs when skin is constantly exposed to the sun and thick, scaly, or crusty areas appear. The scaly or crusty portion is dry and rough. The lesions start out as flat scaly areas and later grow into a tough, wart-like area.

An actinic keratosis site commonly ranges between 2 and 6 millimetres in size, and may be dark or light, tan, pink, red, a combination of all these, or have the same pigment as the surrounding skin. The lesion may appear on any sun-exposed area, such as the face, ears, neck, scalp, chest, backs of hands, forearms, or lips.

Contents [hide]

1 Classification
2 Diagnosis
2.1 Histopathology
3 Prevention
4 Treatment
5 Prognosis
6 Epidemiology
7 Research
8 References
9 External links

Classification

Actinic keratoses may be divided into the following types:[1]

- Hyperkeratotic actinic keratosis
- Pigmented actinic keratosis
- Lichenoid actinic keratosis
- Atrophic actinic keratosis

See also:

- Actinic cheilitis
- Cutaneous horn
- Squamous cell carcinoma in situ

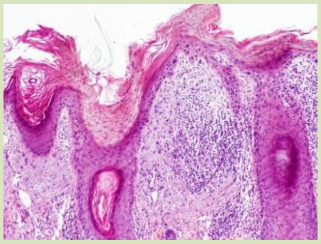

Actinic keratosis, atrophic form

Copyright © by the Association of American Medical Colleges. Unauthorized reproduction is prohibited.

Diagnosis

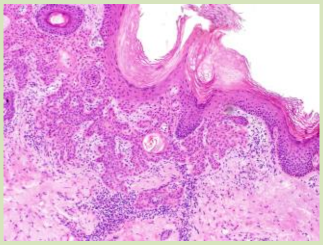

Actinic keratosis, hyperplastic form

Physicians can usually identify actinic keratosis by doing a thorough examination; in principle actinic keratosis is a clinical diagnosis. A biopsy may be necessary when the keratosis is large or thick, to make sure that the lesion is a keratosis and not a skin cancer. Seborrheic keratoses are other lesions that appear in groups as the actinic keratosis do, but are not caused by sun exposure, and are not related to skin cancers. A seborrheic keratosis may be mistaken for an actinic keratosis.

Specialized forms of actinic keratoses include cutaneous horns, in which the skin protrudes in a thick, hornlike manner, and actinic cheilitis, a scaling and roughness of the lower lip and blurring of the border of the lip and adjacent skin.

Histopathology

Actinic keratosis usually shows focal parakeratosis with associated loss of the granular layer of, and thickening of the epidermis. The normal ordered maturation of thekeratinocytes is disordered to varying degrees, there may be widening of the intracellular spaces, and they may also have some cytologic atypia, such as abnormally large nuclei. The underlying dermis often shows severe actinic elastosis and a mild chronic inflammatory infiltrate.[6]

Copyright © by the Association of American Medical Colleges. Unauthorized reproduction is prohibited.

Marked Up Lead Portion of Wikipedia Article after UCSF Medical Student's Last Contribution

Legend

Text added displayed in green

Text moved displayed in orange

Text removed displayed ~~in red strikethrough~~

References added displayed in blue [4]

Hyperlinks added displayed with purple underline

Images added displayed in green circle

Images moved displayed in orange circle

Images deleted displayed with red crosshairs

Actinic keratosis

From Wikipedia, the free encyclopedia

This is an old revision of this page, as edited by Future FamDoc (talk | contribs) at 23:49, 20 November 2014 (*→Research*). The present address (URL) is a permanent link to this revision, which may differ significantly from the current revision.

Actinic keratosis (also called "solar keratosis"[1] and "senile keratosis";[1] abbreviated as "AK") is a pre-cancerous[2] patch of thick, scaly, or crusty skin.[3][4] These growths are more common in fair-skinned people and ~~it is associated with~~ those who are frequently ~~exposed to the~~ in the sun.[5] ~~as it is usually accompanied by solar damage.~~ They usually form when skin gets damaged by ultraviolet (UV) radiation from the sun or indoor tanning beds. AKs ~~The lesions~~ are considered potentially pre-cancerous~~, since some of them progress to squamous cell carcinoma~~; left untreated, they may turn into a type of cancer called squamous cell carcinoma.[4] Untreated lesions have up to a 20% risk of progression to squamous cell carcinoma,[6] so treatment by a dermatologist is recommended.

Progressive ~~D~~development of these ~~lesions~~ growths occur~~s~~ when skin is constantly exposed to the sun over time. ~~and thick, scaly, or crusty areas appear. The scaly or crusty portion is dry and rough.~~ They usually appear as thick, scaly, or crusty areas that often feel dry or rough. ~~The lesions start out as flat scaly areas and later grow into a tough, wart-like area.~~ In fact, AKs are often felt before they are seen,[7] and the texture is often compared to sandpaper. They may be dark, light, tan, pink, red, a combination of all these, or have the same color as the surrounding skin.

An actinic keratosis ~~site~~ lesion commonly ranges between 2 and 6 millimeters in size ~~and may be dark or light, tan, pink, red, a combination of all these, or have the same pigment as the surrounding skin.~~ but can grow to be a few centimeters in diameter.[8] ~~The lesion may~~ They often appear on ~~any~~ sun-exposed areas of the skin, such as the face, ears, neck, scalp, chest, backs of hands, forearms, or lips. Because they are related to sun-damage on the skin, most people who have an AK have more than one.[9]

Diagnosis is made clinically on physical exam, but can be confirmed by looking at cells from the lesion under a microscope. There are various options for treatment, but 5-Fluorouracil cream seems to be popular and effective. By following up with a dermatologist, AKs can be treated before they turn into skin cancer. If skin cancer does develop from an AK lesion, it can be caught early with close monitoring, at a time when treatment can be curative.

Actinic keratosis

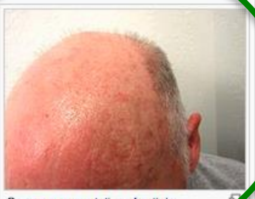

Common presentation of actinic keratosis on a balding head.

Classification and external resources

ICD-10	L57.0
ICD-9	702.0
DiseasesDB	29438
MedlinePlus	000827
Patient UK	Actinic keratosis
MeSH	D055623

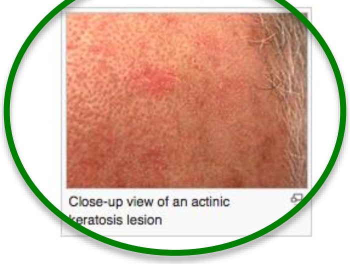

Close-up view of an actinic keratosis lesion

Copyright © by the Association of American Medical Colleges. Unauthorized reproduction is prohibited.

Contents [hide]

1 Signs and symptoms
- 1.1 Clinical variants
- 1.2 Clinical course

2 Cause
- 2.1 Ultraviolet radiation
- 2.2 Skin pigmentation
- 2.3 Balding
- 2.4 Other risk factors

3 Diagnosis
- 3.1 Biopsy
- 3.2 Dermoscopy

4 Prevention

5 Management
- 5.1 Medication
- 5.2 Procedures

6 Prognosis

7 Epidemiology

8 Research

9 References

10 External links

Actinic keratosis

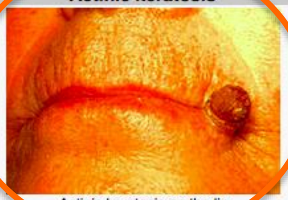

Actinic keratosis on the lip

Classification and external resources	
ICD-10	L57.0
ICD-9	702.0
DiseasesDB	29438
MedlinePlus	000827
Patient UK	Actinic keratosis
MeSH	D055623

Signs and symptoms

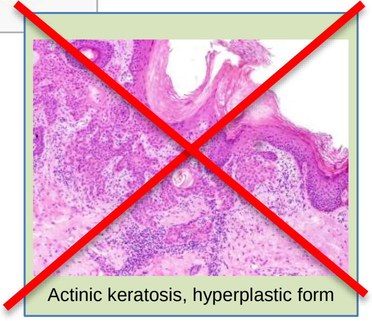

Actinic keratosis, hyperplastic form

Actinic keratoses ("AKs") most commonly present as a white, scaly plaque of variable thickness with surrounding redness; they are most notable for having a sandpaper-like texture when felt with a gloved hand.[10] Skin nearby the lesion often shows evidence of solar damage characterized by notable pigmentary alterations, being yellow or pale in color with areas of hyperpigmentation; deep wrinkles, coarse texture, purpura and ecchymoses, dry skin, and scattered telangiectasias are also characteristic.[11] Photoaging leads to an accumulation of oncogenic changes, resulting in a proliferation of mutated keratinocytes that can manifest as AKs or other neoplastic growths.[8] With years of sun damage, it is possible to develop multiple AKs in a single area on the skin. The lesions are usually asymptomatic, but can be tender, itch, bleed, or produce a stinging or burning sensation.[12] AKs are typically graded in accordance with their clinical presentation: Grade I (easily visible, slightly palpable), Grade II (easily visible, palpable), and Grade III (frankly visible and hyperkeratotic).[13]

Copyright © by the Association of American Medical Colleges. Unauthorized reproduction is prohibited.

Close Up Of Lead Portion of a Wikipedia Article after UCSF Medical Student's Last Contribution

Actinic keratosis

From Wikipedia, the free encyclopedia

This is an old revision of this page, as edited by Future FamDoc (talk | contribs) at 23:49, 20 November 2014 (→*Research*). The present address (URL) is a permanent link to this revision, which may differ significantly from the current revision.

Actinic keratosis

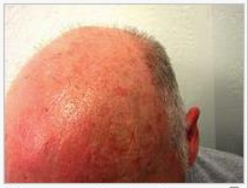

Common presentation of actinic keratosis on a balding head.

Classification and external resources

**ICD-10**	L57.0
**ICD-9**	702.0
**DiseasesDB**	29438
**MedlinePlus**	000827
**Patient UK**	Actinic keratosis
**MeSH**	D055623

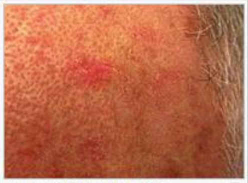

Close-up view of an actinic keratosis lesion

Actinic keratosis (also called "solar keratosis"[1] and "senile keratosis";[1] abbreviated as "AK") is a pre-cancerous[2] patch of thick, scaly, or crusty skin.[3][4] These growths are more common in fair-skinned people and those who are frequently in the sun.[5] They usually form when skin gets damaged by ultraviolet (UV) radiation from the sun or indoor tanning beds. AKs are considered potentially pre-cancerous; left untreated, they may turn into a type of cancer called squamous cell carcinoma.[4]Untreated lesions have up to a 20% risk of progression to squamous cell carcinoma,[6] so treatment by a dermatologist is recommended.

Development of these growths occur when skin is constantly exposed to the sun over time. They usually appear as thick, scaly, or crusty areas that often feel dry or rough. In fact, AKs are often felt before they are seen,[7] and the texture is often compared to sandpaper. They may be dark, light, tan, pink, red, a combination of all these, or have the same color as the surrounding skin. An actinic keratosis lesion commonly ranges between 2 and 6 millimeters in size but can grow to be a few centimeters indiameter.[8] They often appear on sun-exposed areas of the skin, such as the face, ears, neck, scalp, chest, backs of hands, forearms, or lips. Because they are related to sun-damage on the skin, most people who have an AK have more than one.[9]

Diagnosis is made clinically on physical exam, but can be confirmed by looking at cells from the lesion under a microscope. There are various options for treatment, but 5-Fluorouracil cream seems to be popular and effective. By following up with a dermatologist, AKs can be treated before they turn into skin cancer. If skin cancer does develop from an AK lesion, it can be caught early with close monitoring, at a time when treatment can be curative.

Contents [hide]

1 Signs and symptoms
1.1 Clinical variants
1.2 Clinical course
2 Cause
2.1 Ultraviolet radiation
2.2 Skin pigmentation
2.3 Balding
2.4 Other risk factors
3 Diagnosis
3.1 Biopsy
3.2 Dermoscopy
4 Prevention
5 Management
5.1 Medication
5.2 Procedures
6 Prognosis
7 Epidemiology
8 Research
9 References
10 External links

Signs and symptoms

Actinic keratoses ("AKs") most commonly present as a white, scaly plaque of variable thickness with surrounding redness; they are most notable for having a sandpaper-like texture when felt with a gloved hand.[10]Skin nearby the lesion often shows evidence of solar damage characterized by notable pigmentary alterations, being yellow or pale in color with areas of hyperpigmentation; deep wrinkles, coarse texture, purpura andecchymoses, dry skin, and scattered telangiectasias are also characteristic.[11] Photoaging leads to an

Copyright © by the Association of American Medical Colleges. Unauthorized reproduction is prohibited.

accumulation of oncogenic changes, resulting in a proliferation of mutated keratinocytes that can manifest as AKs or other neoplastic growths.[8] With years of sun damage, it is possible to develop multiple AKs in a single area on the skin. The lesions are usually asymptomatic, but can be tender, itch, bleed, or produce a stinging or burning sensation.[12] AKs are typically graded in accordance with their clinical presentation: Grade I (easily visible, slightly palpable), Grade II (easily visible, palpable), and Grade III (frankly visible and hyperkeratotic).[13]

Copyright © by the Association of American Medical Colleges. Unauthorized reproduction is prohibited.
